# Multifrequency Dynamics of Cortical Neuromagnetic Activity Underlying Seizure Termination in Absence Epilepsy

**DOI:** 10.3389/fnhum.2020.00221

**Published:** 2020-06-26

**Authors:** Jintao Sun, Yuan Gao, Ailiang Miao, Chuanyong Yu, Lu Tang, Shuyang Huang, Caiyun Wu, Qi Shi, Tingting Zhang, Yihan Li, Yulei Sun, Xiaoshan Wang

**Affiliations:** Department of Neurology, The Affiliated Brain Hospital of Nanjing Medical University, Nanjing Medical University, Nanjing, China

**Keywords:** childhood absence epilepsy, magnetoencephalography, termination, cerebral cortex, multifrequency

## Abstract

**Purpose:**

This study aimed to investigate the spectral and spatial signatures of neuromagnetic activity underlying the termination of absence seizures.

**Methods:**

Magnetoencephalography (MEG) data were recorded from 18 drug-naive patients with childhood absence epilepsy (CAE). Accumulated source imaging (ASI) was used to analyze MEG data at the source level in seven frequency ranges: delta (1–4 Hz), theta (4–8 Hz), alpha (8–12 Hz), beta (12–30 Hz), gamma (30–80 Hz), ripple (80–250 Hz), and fast ripple (250–500 Hz).

**Result:**

In the 1–4, 4–8, and 8–12 Hz ranges, the magnetic source during seizure termination appeared to be consistent over the ictal period and was mainly localized in the frontal cortex (FC) and parieto-occipito-temporal junction (POT). In the 12–30 and 30–80 Hz ranges, a significant reduction in source activity was observed in the frontal lobe during seizure termination as well as a decrease in peak source strength. The ictal peak source strength in the 1–4 Hz range was negatively correlated with the ictal duration of the seizure, whereas in the 30–80 Hz range, it was positively correlated with the course of epilepsy.

**Conclusion:**

The termination of absence seizures is associated with a dynamic neuromagnetic process. Frequency-dependent changes in the FC were observed during seizure termination, which may be involved in the process of neural network interaction. Neuromagnetic activity in different frequency bands may play different roles in the pathophysiological mechanism during absence seizures.

## Introduction

Childhood absence epilepsy (CAE) is a common pediatric epilepsy syndrome. Children with CAE often suffer absence seizures during which children lose consciousness and stare blankly. Children with CAE have a risk for psychosocial and behavioral comorbidities and may develop persistent absence seizures or other types of epilepsy (Kessler and McGinnis, 2019). CAE mostly occurs in school-age children, ranging from 6 to 12 years of age, and the morbidity of CAE is higher in females than in males.

Childhood absence epilepsy is considered primary generalized epilepsy. On electroencephalography (EEG), seizures are characterized by a highly recognizable pattern of generalized (bilateral, symmetric, and synchronous) 3-Hz spike and wave discharges (SWDs) (Kessler and McGinnis, 2019). However, an increasing number of studies have found that CAE was more likely to have focal brain origins that were responsible for absence seizures (Westmijse et al., 2009; Kim et al., 2011; Tenney et al., 2013; Rozendaal et al., 2016; Kokkinos et al., 2017).

The involvement of the cortex during absence seizures in CAE has been reported in many studies (Tenney et al., 2013; Miao et al., 2014a; Tang et al., 2016; Jun et al., 2019). Moreover, cortical impairment has also been reported even in the interictal period (Xiang et al., 2015b; Qiu et al., 2016; Drenthen et al., 2019). Previous reports on source changes in the pre-ictal period have demonstrated that SWDs built up gradually rather than occurring suddenly and that the cortex played a leading role in the initiation of absence seizures (Gupta et al., 2011; Jacobs-Brichford et al., 2014; Wu et al., 2017b).

Although the changes in brain activity before the onset of absence seizures have been intensively investigated, the characteristics of neural activity underlying the termination of absence seizures remain poorly understood. Similar to the onset of absence seizures, the termination of absence seizures is well known to be an important part of the evolution of absence seizures, and knowledge of the features of seizure termination may help to further understand the potential self-regulatory mechanism for absence seizures, develop novel therapeutic strategies, and eventually improve the long-term prognosis of CAE. Previous studies focusing on the termination of seizures were mainly conducted at the cellular and energy metabolism levels (Lado and Moshe, 2008; Boison and Steinhauser, 2018; Kovacs et al., 2018). With the progress of technology, neuroimaging has provided new insight for the study of seizure termination. Recently, several studies have reported that the cortex was involved in the termination of SWDs (Benuzzi et al., 2015; Sysoeva et al., 2016; Jiang et al., 2019). However, the specific cortical regions involved in the termination of absence seizures are unclear.

Magnetoencephalography (MEG) is a non-invasive imaging technique that can be used to detect neuromagnetic signals from the brain. MEG and EEG have a similar high temporal resolution. However, MEG signals are usually unaffected by the skull and skin, which leads to a higher spatial resolution than that of EEG (Babiloni et al., 2009). Several studies have reported that MEG can localize epileptiform activity more accurately than EEG and provide reliable localization in the brain cortex (Alkawadri et al., 2018; Tamilia et al., 2019; van Klink et al., 2019). Moreover, MEG has been increasingly used in the evaluation of epileptogenic foci before epileptic surgery because of its non-invasive precise localization (De Tiege et al., 2017; Tamilia et al., 2017). In addition, the advantage over functional magnetic resonance imaging (fMRI) is that MEG can measure at millisecond temporal resolution, which renders it an ideal tool for the investigation of multifrequency epileptic activities (Moradi et al., 2003; Babiloni et al., 2009).

The present study was undertaken using MEG to analyze cortical neuromagnetic activity from 18 CAE patients from low- to high-frequency bands to investigate whether a pattern of specific cortical neural activity exists during the termination of absence seizures, which may be different from that during the ictal period.

## Materials and Methods

Eighteen patients with CAE were recruited from the Nanjing Brain Hospital and Nanjing Children’s Hospital. The inclusion criteria were as follows: (1) a clinical diagnosis of CAE in line with the International League Against Epilepsy Proposal for Revised Classification of Epilepsies and Epileptic Syndromes; (2) typical bilateral, synchronous, symmetrical, approximately 3-Hz SWDs on a normal background in EEG with an ictal duration ≥3 s; (3) no history of receiving antiepileptic medication; and (4) no abnormal MRI results. The exclusion criteria were as follows: (1) CAE coexisting with other types of epilepsy or disease and (2) the presence of metal implants in the head. The medical ethics committees of Nanjing Children’s Hospital, Nanjing Brain Hospital, and Nanjing Medical University provided permission for the present study. Informed assent was signed by all subjects, and consent was received from parents/guardians. All methods were performed in accordance with the relevant guidelines and regulations of the Declaration of Helsinki for human experimentation.

### Magnetoencephalography Recordings

Magnetoencephalography data from all children with CAE acquired using a whole-head CTF 275 Channel MEG system (VSM Medical Technology Company, Canada) were recorded in a magnetically shielded room at the MEG Center of Nanjing Brain Hospital. Before MEG recording, three coils were affixed to the nasion and the left and right pre-auricular points of each patient to fix the head location in the MEG system. All subjects were told to remain still with their eyes closed during MEG recording. For each subject, the MEG recording duration was 120 s. At least six MEG data recordings were acquired for each subject with CAE, which ensured that complete and usable ictal data were available for further study. When the head movement was ≥5 mm or any artifact was present in the MEG data, the data were considered invalid and were recorded again. If ictal data were not captured by MEG recording, another MEG recording was performed, and the subjects were told to hyperventilate to provoke additional absence seizures. The rate of sampling for data acquisition was 6,000 Hz with noise cancelation of third-order gradients during the MEG recordings.

### MRI Scan

Three-dimensional structural images were obtained by a 3.0-T MRI scanner (Siemens, Germany). Anatomic 3D T1-weighted images were obtained using a rapid gradient echo sequence (TR/TE = 1,900/2.48 ms). The imaging parameters were as follows: the field of view was 250 × 250 mm; the flip angle was 9°; and the matrix was 512 × 512. For each subject, 176 sagittal slices were collected. All subjects were instructed to minimize their head movements during scanning procedure, whereas markers were placed on the same three fiducial positions used for MEG to co-register the imaging data with the MEG data. All anatomical landmarks digitized in the MEG study were identifiable in the MRI.

### Data Analysis

Magnetoencephalography data were first visually inspected for removing artifacts and motion-related noise. Then, visually inspected MEG data were band-pass filtered with 1–4 Hz bandwidth. The ictal segments were selected by identifying 3-Hz SWDs. We assessed the ictal segments using an audio-visual system in which we observed behavioral changes during absence seizures, such as staring, in the subjects with CAE. The first spike wave in SWDs was defined as the onset of a seizure, and the last slow wave component of SWDs was defined as the termination of the seizure. Epileptic waveforms were identified by two experienced epileptologists, and the interrater reliability was satisfactory for the identification of epileptic waveforms.

**FIGURE 1:**
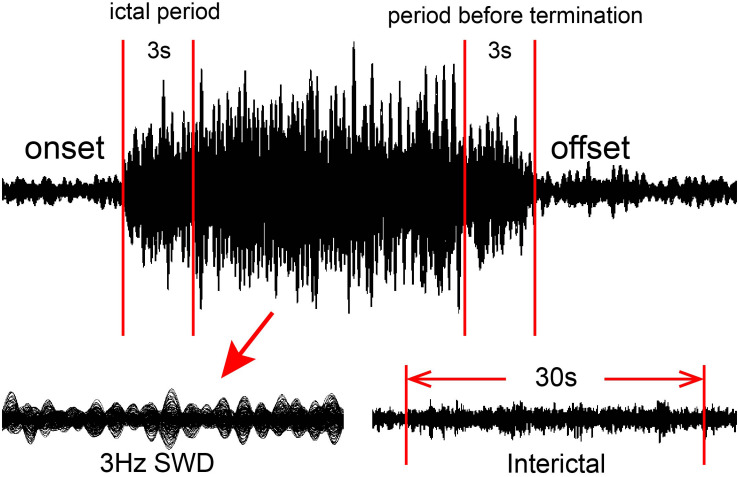
Schematic of magnetoencephalography (MEG) waveform analysis. MEG data were recorded from 18 patients. For each eligible seizure, two 3-s segments were selected as the ictal period and the period during termination of the seizure, whereas one 100-s corresponding segment was selected as the interictal period.

A total of two segments with a length of 3 s were selected to represent the ictal period and the period during termination of the seizure. Waveforms with a 10-s time window away from the ictal segment at least 30 s were considered interictal control data for further analysis (Figure 1).

The segments selected from the MEG data were analyzed in the following seven frequency bands: delta (1–4 Hz), theta (4–8 Hz), alpha (8–12 Hz), beta (12–30 Hz), gamma (30–80 Hz), ripple (80–250 Hz), and fast ripple (250–500 Hz). To eliminate power-line noise from the MEG data, notch filters for 50 Hz and its harmonics were applied.

Neuromagnetic sources were analyzed with an individual MRI-based head model. To objectively assess seizure-related epileptic activity in the brain, the whole brain was scanned with 3-mm resolution (115,136–115,562 voxels, depending on the size of the brain) (Xiang et al., 2014). Volumetric source imaging was computed for each frequency band for each subject. Detailed mathematical algorithms and validations have been described recently (Xiang et al., 2014, 2015a). To quantify source strength and reliability, each voxel in the source imaging comprised more than one parameter (multiple parameters per position). Specifically, each voxel had one parameter to represent the confidence volume and another parameter to describe the spectral power. The confidence volume was used for quality control: if the source voxel had a confidence volume smaller than 5 mm × 5 mm × 5 mm, the source voxel was considered reliable; otherwise, the source voxel was considered unreliable. Because the source strength was statistically analyzed (Xiang et al., 2015a; Shi et al., 2019), no units for the source measurements were provided. MEG source imaging was co-registered to MRI with the three fiducial points that had been placed prior to the MEG study and then spatially normalized for group analyses (Gilbert et al., 2012). Similar to a previous report (Xiang et al., 2009b), we performed comparisons at the source levels.

The definition of accumulated source imaging (ASI) was the volumetric summation of source activity over a period of time. The sources were localized by ASI using node-beam lead fields (Xiang et al., 2014). Because each node-beam lead field represented a form of either source-beamformer or subspace solution, the ASI had multiple source beamformers to separate correlated sources.

### Statistical Analysis

Fisher’s exact test was utilized to assess the predominant source localization between different periods in each frequency band. Student’s *t*-test was applied to compare changes in source strength between different periods in each frequency band. Partial correlation analysis was used to estimate correlations between clinical features and the peak source strength after adjustment for age and sex. The *p*-value threshold in our study was 0.05. For multiple comparisons, Bonferroni correction was applied. All statistical analyses were performed using SPSS 19.0 for Windows (SPSS Inc., Chicago, IL, United States).

## Results

### Clinical Characteristics

Eighteen drug-naive patients with CAE were enrolled in this study (age range, 5–11 years; mean age, 8.4 ± 1.75 years; sex, 5 males and 13 females). The average course of epilepsy was 10.2 ± 7.60 months. The average seizure frequency was 6.6 ± 4.0 times/day. The clinical features of the subjects with CAE are shown in Table 1. A total of 33 ictal segments with an average ictal duration of 14.1 s (range, 6–36 s) were detected in the 18 subjects with CAE during MEG recording for the following analysis.

**TABLE 1:** Characteristics of the patients with CAE.

Patient	Sex (F/M)	Age (years)	Duration of disease (months)	Frequency of seizures (times/day)	Time between diagnosis and the MEG test (day)
1	M	10	5	6	0
2	F	7	5	3	1
3	F	6	5	2–3	0
4	M	8	5	10	0
5	M	8	6	7	0
6	F	5	6	2–3	0
7	F	10	12	5	0
8	F	9	16	5–6	1
9	F	10	11	5	0
10	F	10	12	6–7	0
11	F	11	23	8	0
12	F	10	32	8	0
13	F	5	3	8	1
14	F	8	8	8	0
15	F	8	4	20	0
16	F	9	12	4–5	0
17	M	8	4	5	0
18	M	9	15	7–8	0

*CAE, childhood absence epilepsy; MEG, magnetoencephalography; F, female; M, male.*

### Source Localization Pattern

**FIGURE 2:**
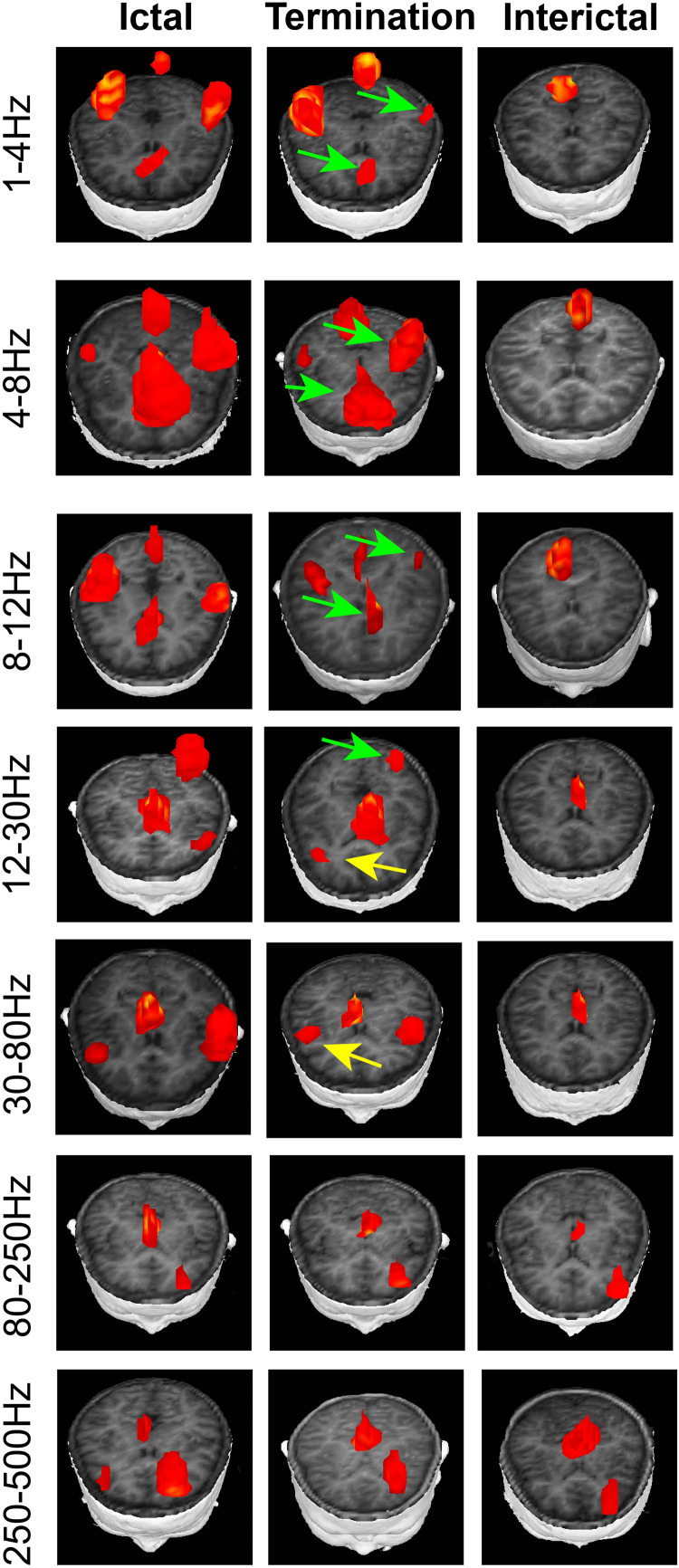
Typical distribution of neuromagnetic source localization in the three periods in the seven frequency bands recorded from patients with CAE. In the 1–4, 4–8, 8–12, and 12–30 Hz ranges, the magnetic source during seizure termination mainly localized in the frontal cortex (FC) and parieto-occipito-temporal junction (POT) compared with that during the interictal period. In the 12–30 and 30–80 Hz ranges, a significant difference was identified in the source location in the FC during seizure termination compared with the ictal and interictal periods. Green arrows indicate the locations of neuromagnetic sources, which show significant differences between the period during seizure termination and the interictal period. Yellow arrows point to the regions that statistically changed during seizure termination compared with the ictal and interictal periods.

**FIGURE 3:**
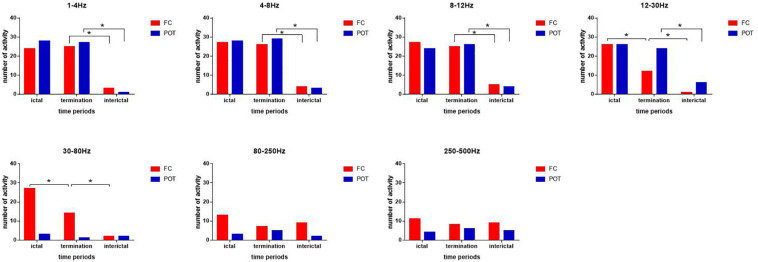
Changes in the number of neuromagnetic source activities in the three periods for the seven frequency bands. The number of source locations is listed on the *y*-axis. Three periods are located on the *x*-axis. *p < 0.05 after Bonferroni correction.

In the 1–4, 4–8, 8–12, and 12–30 Hz ranges, the magnetic source during seizure termination was mainly localized in the frontal cortex (FC) and parieto-occipito-temporal junction (POT) compared with that during the interictal period, which appeared to be consistent with the localization in the ictal period. No significant change in magnetic source localization in the POT was observed during seizure termination (Figures 2, 3).

In the 12–30 and 30–80 Hz ranges, the source was mainly localized in the FC and deep brain area (DBA) in the ictal period. A significant difference in the source location in the FC was identified during seizure termination compared with the ictal and interictal periods (*p* < 0.05) (Figures 2, 3).

In the 80–250 and 250–500 Hz ranges, the source was mainly localized in the FC and DBA. No significant difference was detected in the magnetic source location over all three periods (Figures 2, 3).

### Peak Source Strength of Neuromagnetic Activity

**FIGURE 4:**
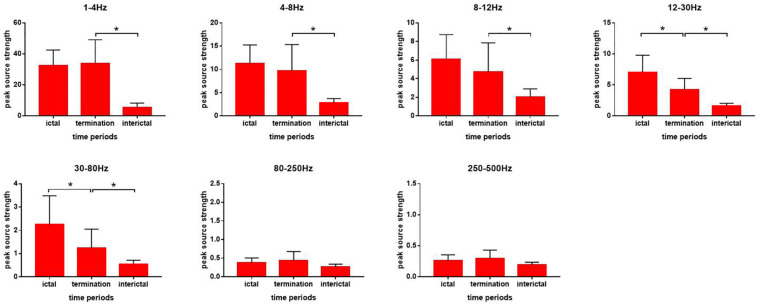
Changes in the strength of neuromagnetic activity in the seven frequency bands during absence seizures. Source strength measurements are shown on the *y*-axis. Three periods are listed on the *x*-axis. *p < 0.05 after Bonferroni correction.

In the 1–4, 4–8, and 8–12 Hz ranges, the peak source strength during seizure termination was significantly higher than that during the interictal period (*p* < 0.05). No significant difference in peak source strength was found between the ictal period and during seizure termination (Figure 4).

In the 12–30 and 30–80 Hz ranges, the peak source strength during seizure termination decreased significantly compared with that during the ictal period and was still significantly higher than that during the ictal period (*p* < 0.05) (Figure 4).

In the 80–250 and 250–500 Hz ranges, no significant difference in peak source strength was found over all three periods (Figure 4).

### Clinical Correlations

**FIGURE 5:**
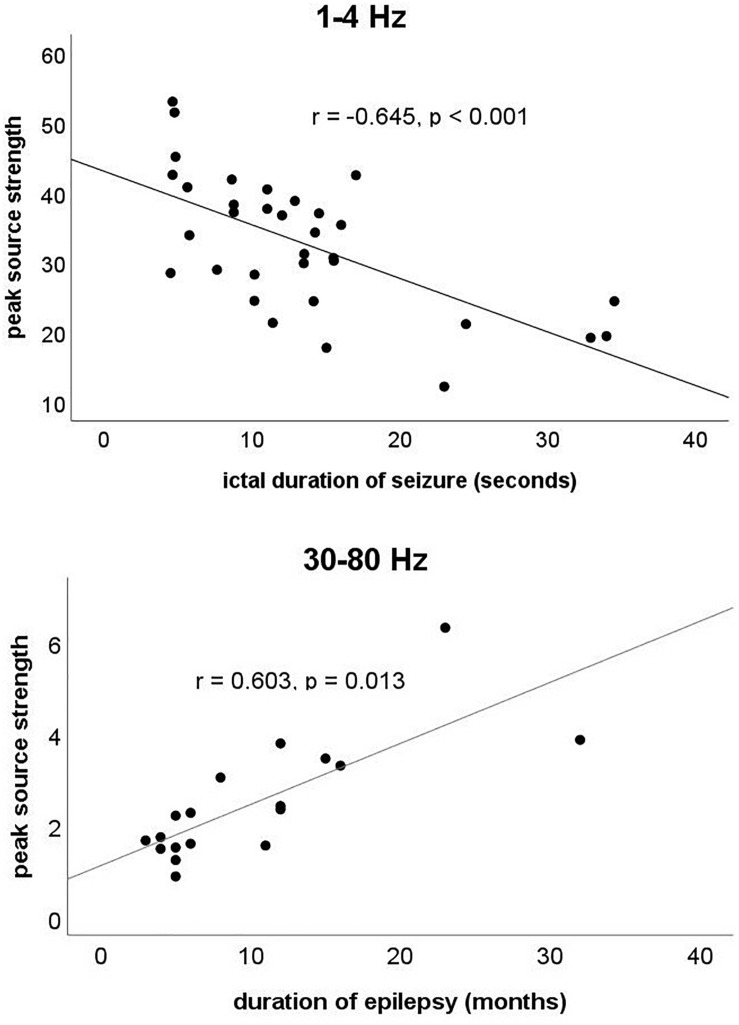
The *y*-axis represents the ictal peak source strength. The *x*-axis at 1–4 Hz represents the ictal duration of the seizure, whereas at 30–80 Hz, the *x*-axis represents the duration of epilepsy. Partial correlation analysis showed that the ictal peak source strength in the 1–4 Hz range was negatively correlated with the ictal duration of the seizure (*r* = –0.645, *p* < 0.001) after adjustment for age and sex, whereas in the 30–80 Hz range, it was positively correlated with the duration of epilepsy (*r* = 0.603, *p* = 0.013) after adjustment for age and sex.

As shown in Figure 5, our data demonstrated that during the ictal period, the ictal peak source strength in the 1–4 Hz range was negatively correlated with the ictal duration of the seizure (*r* = −0.645, *p* < 0.001) after adjustment for age and sex, whereas in the 30–80 Hz range, it was positively correlated with the duration of epilepsy (*r* = 0.603, *p* = 0.013) after adjustment for age and sex. No significant correlation was found in the other frequency bands.

## Discussion

Our study investigated cortical neuromagnetic activity in subjects with CAE from low- to high-frequency ranges using MEG. The neuromagnetic activity revealed significant frequency-dependent differences during seizure termination compared with the ictal and interictal periods. The ictal peak source strength of neuromagnetic activity in specific frequency bands was correlated with the ictal duration of the seizure and the duration of epilepsy.

Most previous studies have suggested that the FC is more likely to play a critical role in the initiation and propagation of SWDs (Holmes et al., 2004; Szaflarski et al., 2010; Tenney et al., 2013; Miao et al., 2014a; Tang et al., 2016; Wu et al., 2017b). In the present study, we found lower activity in the FC during the termination of absence seizures than that during the ictal period, suggesting that the activity of the FC decreases significantly during the termination of absence seizures. An EEG-fMRI study demonstrated that the blood oxygenation level-dependent (BOLD) signal was decreased in the FC at seizure termination, indicating that the FC may be related to the termination of absence seizures (Benuzzi et al., 2015). Furthermore, our results showed that the decrease in activity in the FC mainly occurred in the 12–30 and 30–80 Hz frequency bands. In other words, we found a frequency-dependent neuromagnetic activity pattern during the termination of absence seizures. A previous study revealed a frequency-dependent feature of the ictal network in absence epilepsy, which may partially explain our results (Tenney et al., 2014, 2018). Similarly, another study reported that SWDs revealed alternating ictal patterns between localization during the spikes and generalization during the waves (Ossenblok et al., 2019). In addition, several studies have shown that part of the spike wave during absence seizures was generated by the FC (Clemens et al., 2007; Jun et al., 2019). Another study also reported that the first spike wave of SWDs was localized in the lateral FC during absence seizures (Tenney et al., 2013). Therefore, we speculate that the decrease in activity in the FC in the 12–30 and 30–80 Hz ranges may be related to decreased activity in the spike wave during seizure termination. Furthermore, our results suggest that the termination of absence seizures was a gradual process rather than a sudden event, which in line with previous studies (Luttjohann et al., 2014; Jiang et al., 2019).

The present study showed that the magnetic source locations in high-frequency ranges (80–250 and 250–500 Hz) were mainly in the FC and DBA. No significant difference in magnetic source location was observed over all three periods, suggesting that the pattern of neural activity in high-frequency bands differed from that in all other frequency bands (<80 Hz), which is supported by recent studies (Tang et al., 2016; Wu et al., 2017b; Jiang et al., 2019). In recent years, increasing research on high-frequency oscillations (HFOs) has been presented, and HFOs have been considered a new biomarker for epilepsy (Xiang et al., 2009a, 2010; Miao et al., 2014b; Tang et al., 2016). HFOs have been reported to be correlated with the pathophysiology of epilepsy and used to localize the epileptogenic zone in epileptic surgery (Haegelen et al., 2013; Kerber et al., 2014). Previous reports have demonstrated that source localization in high-frequency bands was more focal and stable than that in low-frequency bands (Miao et al., 2014b; Tang et al., 2016; Wu et al., 2017b). Furthermore, a study found that HFOs could precede the onset of a seizure and persist after the postictal period (Fuertinger et al., 2016). These related studies suggested the existence of different cellular and network mechanisms between HFOs and other neural activity (Draguhn et al., 1998; Traub et al., 2003; Roopun et al., 2010). HFOs are suited to interact with neighboring neurons, and lower frequencies are suitable for integration over a wider range (Sauseng and Klimesch, 2008; Xiang et al., 2015b; Tang et al., 2016; Wu et al., 2017a, b; Tenney et al., 2018). Therefore, we speculate that the activity of the FC in high-frequency bands may be related to the generation of absence seizures. Nevertheless, the specific function of HFOs still requires further investigation.

Our study found that the ictal peak source strength at 30–80 Hz was positively correlated with the duration of epilepsy. Previous studies have suggested that gamma oscillations were produced by the activity of fast-spiking inhibitory interneurons (Cardin et al., 2009; Sohal, 2012). One possible explanation for the correlation between peak source strength and the duration of epilepsy is that long-term chronic neuronal structural damage may be the structural basis for the increase in peak source strength caused by frequent abnormal discharges during the ictal period (Tang et al., 2016). In addition, we also found that the ictal peak source strength at 1–4 Hz was negatively correlated with the ictal duration of the seizure. Delta oscillations were mainly produced by cortical pyramidal neurons (Neckelmann et al., 2000; Cardin et al., 2009). Abnormal delta oscillations were found to be associated with impairment of consciousness during epileptic seizures, which may be due to an inhibitory effect on the excitability of the cortex (Shmuel et al., 2002, 2006; Pittau et al., 2013; Holler and Trinka, 2015; Unterberger et al., 2018). However, another study revealed that low-frequency oscillations could also inhibit neural activity in higher-frequency bands and promote seizure termination (Medvedev, 2002; Tenney et al., 2014). An interaction between different frequency bands may be achieved through cross-frequency coupling as reported by a previous study, which requires further investigation (Aru et al., 2015). Although our results are not sufficient to prove the specific mechanism of the correlation between peak source strength in different frequency bands and clinical features, we speculate that neural activity in different frequency bands may play different roles in absence epilepsy.

### Limitations

Several limitations to our research exist. First, because absence seizures are unpredictable, collecting complete ictal data is difficult, resulting in a small sample size. However, we applied strict criteria and excluded other forms of idiopathic generalized epilepsy to establish a strictly homogeneous group of patients in terms of diagnosis for this study despite the limited sample size. Second, although recent studies have certified that MEG can detect the DBA (Papadelis et al., 2009; Leiken et al., 2014), the spatial resolution in the DBA on MEG remains debatable. In several studies, although the activities of the DBA were mainly regarded as the activities of the thalamus (Wu et al., 2017b; Jiang et al., 2019), we cannot rule out the possibility of artifacts and noise, which may disturb our results. Therefore, we removed the analysis and discussions of neuromagnetic activities related to the DBA in this study. This issue can be resolved with the advancement of MEG technology in the future. Third, the 3-s time windows selected in this study were not sufficiently short, preventing us from observing changes in neuromagnetic activity within 3 s during the termination of absence seizures. Further investigations with shorter time windows are essential in the future. Fourth, although we recorded MEG signals under the same experimental conditions and minimized artifacts using accumulated technology and other measures, artifacts from electromyography, magnetocardiography, and other signals may still be included in our results. More investigations in the future are required to determine whether artifacts are completely eliminated. Finally, the source imaging technology of software is limited and not wholly reliable. Thus, our results must be verified using other brain imaging software in future investigations.

## Conclusion

In conclusion, we demonstrated that the termination of absence seizures is associated with a dynamic neuromagnetic process and that frequency-dependent changes in the activity of the FC during termination of absence seizures may be involved in the process of seizure termination. The ictal peak source strength in the 1–4 Hz range was negatively correlated with the ictal duration of the seizure, whereas in the 30–80 Hz range, it was positively correlated with the duration of epilepsy, suggesting that neuromagnetic activity in different frequency bands may play different roles in the pathophysiological mechanism of CAE. The specific mechanism of neural activity in multifrequency bands underlying seizure termination needs to be investigated further.

## Data Availability Statement

The raw data supporting the conclusions of this article will be made available by the authors, without undue reservation, to any qualified researcher.

## Ethics Statement

The studies involving human participants were reviewed and approved by the medical ethics committees of Nanjing Brain Hospital, the medical ethics committees of Nanjing Children’s Hospital and the medical ethics committees of Nanjing Medical University. Written informed consent to participate in this study was provided by the participants’ legal guardian/next of kin. Written informed consent was obtained from the minor(s)’ legal guardian/next of kin for the publication of any potentially identifiable images or data included in this article.

## Author Contributions

JS, YG, AM, and XW designed the research. JS, QS, AM, LT, and SH analyzed the data. YG, CY, TZ, YL, YS, and CW recruited the participants and acquired the images. JS wrote the manuscript. XW revised the manuscript. All authors approved the final submitted version and agreed to be accountable for its content.

## Conflict of Interest

The authors declare that the research was conducted in the absence of any commercial or financial relationships that could be construed as a potential conflict of interest.
